# Identifying and Evaluating Field Indicators of Urogenital Schistosomiasis-Related Morbidity in Preschool-Aged Children

**DOI:** 10.1371/journal.pntd.0003649

**Published:** 2015-03-20

**Authors:** Welcome M. Wami, Norman Nausch, Nicholas Midzi, Reggis Gwisai, Takafira Mduluza, Mark Woolhouse, Francisca Mutapi

**Affiliations:** 1 Institute of Immunology and Infection Research, School of Biological Sciences, University of Edinburgh, Edinburgh, United Kingdom; 2 Centre for Immunity, Infection and Evolution, School of Biological Sciences, University of Edinburgh, Edinburgh, United Kingdom; 3 National Institute of Health Research, Causeway, Harare, Zimbabwe; 4 Ministry of Health and Child Care, Murewa District Hospital, Murewa, Zimbabwe; 5 Department of Biochemistry, University of Zimbabwe, Mount Pleasant, Harare, Zimbabwe; Ministère de la Santé Publique et de la Lutte contre les Endémies, NIGER

## Abstract

**Background:**

Several studies have been conducted quantifying the impact of schistosome infections on health and development in school-aged children. In contrast, relatively little is known about morbidity levels in preschool-aged children (≤5 years) who have been neglected in terms of schistosome research and control. The aim of this study was to compare the utility of available point-of-care (POC) morbidity diagnostic tools in preschool *versus* primary school-aged children (6–10 years) and determine markers which can be used in the field to identify and quantify *Schistosoma haematobium*-related morbidity.

**Methods/Principal Findings:**

A comparative cross-sectional study was conducted to evaluate the performance of currently available POC morbidity diagnostic tools on Zimbabwean children aged 1–5 years (n=104) and 6–10 years (n=194). Morbidity was determined using the POC diagnostics questionnaire-based reporting of haematuria and dysuria, clinical examination, urinalysis by dipsticks, and urine albumin-to-creatinine ratio (UACR). Attributable fractions were used to quantify the proportion of morbidity attributable to S. haematobium infection. Based on results of attributable fractions, UACR was identified as the most reliable tool for detecting schistosome-related morbidity, followed by dipsticks, visual urine inspection, questionnaires, and lastly clinical examination. The results of urine dipstick attributes showed that proteinuria and microhaematuria accounted for most differences between schistosome egg-positive and negative children (*T*=-50.1; p<0.001). These observations were consistent in preschool vs. primary school-aged children.

**Conclusions/Significance:**

Preschool-aged children in endemic areas can be effectively screened for schistosome-related morbidity using the same currently available diagnostic tools applicable to older children. UACR for detecting albuminuria is recommended as the best choice for rapid assessment of morbidity attributed to *S*. *haematobium* infection in children in the field. The use of dipstick microhaematuria and proteinuria as additional indicators of schistosome-related morbidity would improve the estimation of disease burden in young children.

## Author Summary

Schistosomiasis is a major parasitic disease affecting children in Africa, with impacts on health, growth and cognitive development. Recently, the World Health Organization has recommended inclusion of preschool-aged children (≤5 years) in schistosome control programmes. However, so far the performance of available morbidity diagnostic tools has not been thoroughly evaluated in this age group. To address this knowledge gap, we conducted a study in preschool children comparing the utility of currently available point-of-care tools for diagnosing *Schistosoma haematobium*-related morbidity, namely: questionnaire-reported haematuria and dysuria, clinical examination, dipstick urinalysis, and measurement of urine albumin-to-creatinine ratio (UACR). We also investigated the performance of these tools in older children (6–10 years). Our study identified UACR as the most reliable tool for detecting schistosome-related morbidity in terms of the morbidity attributable to schistosome infection, followed by dipsticks, visual urine inspection, questionnaires, and lastly clinical examination The study further showed that the tools currently used in school-aged children for diagnosing schistosome-related morbidity can be extended to preschool children, allowing easier integration of this age group into treatment and monitoring programmes.

## Introduction

Urogenital schistosomiasis is a major parasitic disease caused by *Schistosoma haematobium* affecting children in Africa, with negative impacts on child health, growth and cognitive development [1]. Chronic infection with the parasite can cause anaemia, malnutrition, and organ complications such as bladder fibrosis and kidney failure [2]. Schistosome control programmes focus on preventive chemotherapy with the antihelminthic drug of choice, praziquantel, to reduce or prevent the development of severe morbidity due to schistosome infection, and thereby improving health of the infected individuals and communities [3]. In order to achieve these goals and evaluate the effects of control programmes, an understanding of the morbidity due to schistosome infection is essential [4]. This requires the use of reliable rapid diagnostic tools that can be used in the field [5].

In recent years progress has been made towards improving methods for measuring *S*. *haematobium*-related morbidity and various techniques have been evaluated in older children and adult populations [5]. For example, ultrasonography has been shown to be effective in detecting organ-specific morbidity [6,7]. However, the need for specialized equipment and trained personnel reduces its utility for large population studies in the field. Urinalysis has been used as a rapid indirect assessment tool for early urinary tract morbidity due to schistosomiasis [8]. In addition, standardized questionnaires recommended by the WHO for rapid screening of *S*. *haematobium* infection and morbidity have been extensively used in endemic regions [9]. Most of these studies have focused on older children, typically primary school-aged children (6–10 years), or older individuals.

The WHO has recently recommended the inclusion of preschool children (aged 5 years and below) in schistosome control programmes [10], but the performance of the currently available point-of-care (POC) diagnostic tools for detecting schistosome-related morbidity have not yet been systematically evaluated in this age group. In addition, the utility of these POC tools has not been compared in a single study between preschool and primary school-aged (6–10 years) children, who are the current main targets of schistosome control programmes. Measuring the burden of schistosome disease in the whole population, including preschool children is important for the assessment of the effectiveness of control programmes and thus their prioritization and sustenance in affected countries (often with limited health budgets). Although extensive work has been done and a few recent studies published on morbidity due to *S*. *mansoni* infection in preschool children [11], to date there is still a paucity of studies quantifying the burden of *S*. *haematobium*-related morbidity in preschool children and the applicability of current POC morbidity diagnostics in these young children has not been extensively evaluated. To address this knowledge gap, we conducted a study in preschool and primary school children endemically exposed to *S*. *haematobium infection* assessing the utility of available diagnostic tools in identifying POC markers of schistosome-related morbidity.

The first aim of the study was to characterise the morbidity in the children detected using the available POC tools. Since the morbidity markers currently used are general as opposed to being schistosome specific, they may detect morbidity unrelated to schistosome infection. Therefore, the second aim of the study was to relate the measures of morbidity to schistosome infection and determine the fraction of morbidity attributable to schistosome infection. The overall results would allow us to determine if POC diagnostics available for use in primary school-aged children can be reliably used in the field to quantify and monitor levels of morbidity attributable to *S*. *haematobium* infection in young children aged 5 years and below.

## Materials and Methods

### Ethical statement

Ethical and institutional approval for the study was obtained from the Medical Research Council of Zimbabwe and the University of Zimbabwe, respectively. Permission to conduct the study was received from the Provincial Medical Director, the District Educational Officer, and Heads of schools in the study area. Study aims and procedures were explained to participants, and their parents/guardians in the local language, Shona. Prior to enrolment of study participants, written informed consent was obtained from parents/guardians and oral assent obtained from children. The children were recruited into the study on voluntary basis and were free to withdraw at any time with no further obligation. After sample collection, participants were offered treatment with the standard dose of 40 mg/kg praziquantel, administered by the local physician. The praziquantel drug was procured from a local supplier (Pharmaceutical and Chemical Distributors (Pvt) Ltd, Harare, Zimbabwe), registered and licensed to sell the drug in Zimbabwe.

### Study area

The cross-sectional study was conducted in Murewa district, in the north-east of Zimbabwe (31°90'E; 17°63'S) where *S*. *haematobium* is endemic. Prevalence of *S*. *mansoni* was low (<10%) [9] in this current study population as previously reported in other studies conducted in the same area [12,13]. There were no soil-transmitted helminths infections detected in this study population.

### Participants

Children aged 1–10 years were recruited from crèches, early child development centres, and local primary schools between February 28, and March 09, 2012. To be included in this study, participants had to meet the following criteria: (1) been lifelong residents of the study area, (2) had no prior history of antihelminthic treatment (assessed by questionnaires administered to parents/guardians for all children), and (3) provided at least two urine, and two stool samples for parasitological examinations on consecutive days. The exclusion criteria were: (1) presenting with clinical symptoms of tuberculosis or malaria/fever, (2) recent major illness/operation, and (3) diagnosed positive for soil-transmitted helminths. None of the children were excluded based on these criteria.

### Parasitology and serology

*S*. *haematobium* infection was determined by microscopic enumeration of eggs in urine processed using the standard urine filtration method [14]. Children were classified as infected if at least one parasite egg was detected in any of their urine samples collected on consecutive days. Infection intensity was defined as the arithmetic mean egg counts/10 mL of at least two urine samples collected on three consecutive days. Stool samples were processed using the Kato-Katz method, with duplicate thick smears (41.7 mg) performed per sample [15], and subsequent egg enumeration by microscopy for the diagnosis of *S*. *mansoni* and soil-transmitted helminths. Children were designated infected with *S*. *mansoni* or soil-transmitted helminths if at least one parasite egg was detected in any of the two stool samples collected on consecutive days. A small proportion, 6.0% (n = 18) of the children in our study was found positive for S. mansoni. We compared the morbidity characteristics of these children to those of a random sample drawn from age and sex matched S. mansoni negative children and no differences were observed, hence these children were kept in our study for the final analyses. None of the children in this study were found positive for STHs.

We have recently shown that egg count lacks sensitivity in diagnosing light schistosome infections in children [16]. Thus, in addition to parasitology, IgM antibody responses directed against soluble egg antigens (SEA) were used to improve the diagnosis of *S*. *haematobium* infection. Details of the protocols used to quantify the serum antibody levels are published elsewhere [17]. Children were categorized as infected based on serology if their anti-egg IgM antibody levels were more than two standard deviations above the mean estimated from sera of negative controls, as outlined in our recently published study [16].

### Morbidity measurement

#### Urinalysis

Urine samples collected on the first day of the survey were examined for visible haematuria. Uristix reagent strips (Uripath, Plasmatec, UK) were used to test for the presence of nitrites, leucocytes, blood (microhaematuria), proteins (proteinuria), and physical characteristics (pH, specific gravity). To assess observer bias in dipstick readings, a random sample of 102 of the 298 urine samples was further tested using the Multistix 10SG (Bayer, UK), and the results read automatically using Siemens' CLINITEK Status+ Analyzer (Bayer, UK). For all the attributes tested, a high proportion of overall agreement (p_overall_>60.0%) between the two dipstick tests was noted, showing no evidence of significant observer effect. CLINITEK Microalbumin Reagent Strips (Bayer, UK) were used to determine urine albumin-to-creatinine ratio (UACR) threshold levels on first day urine samples. Following manufacturer’s guidelines, high-abnormal UACR (>33.9 mg/mmol) was used to ascertain presence of albuminuria [18], a biological marker of urinary tract infection and an early predictor of progressive kidney disease [8].

#### Questionnaires

A pre-tested questionnaire on recent/current presence of haematuria and dysuria, constructed in English and translated to the local language, Shona, was administered to parents/guardians of preschool-aged children. An alternative version of the questionnaire was administered to the primary school-aged children.

#### Clinical examination

All participants underwent a non-intrusive clinical examination, involving abdominal palpation, conducted by experienced study clinicians to determine current health status and schistosome-related anomalies (e.g., epigastric or abdominal pains).

### Statistical methods

#### Sample size calculation

**Fig 1:**
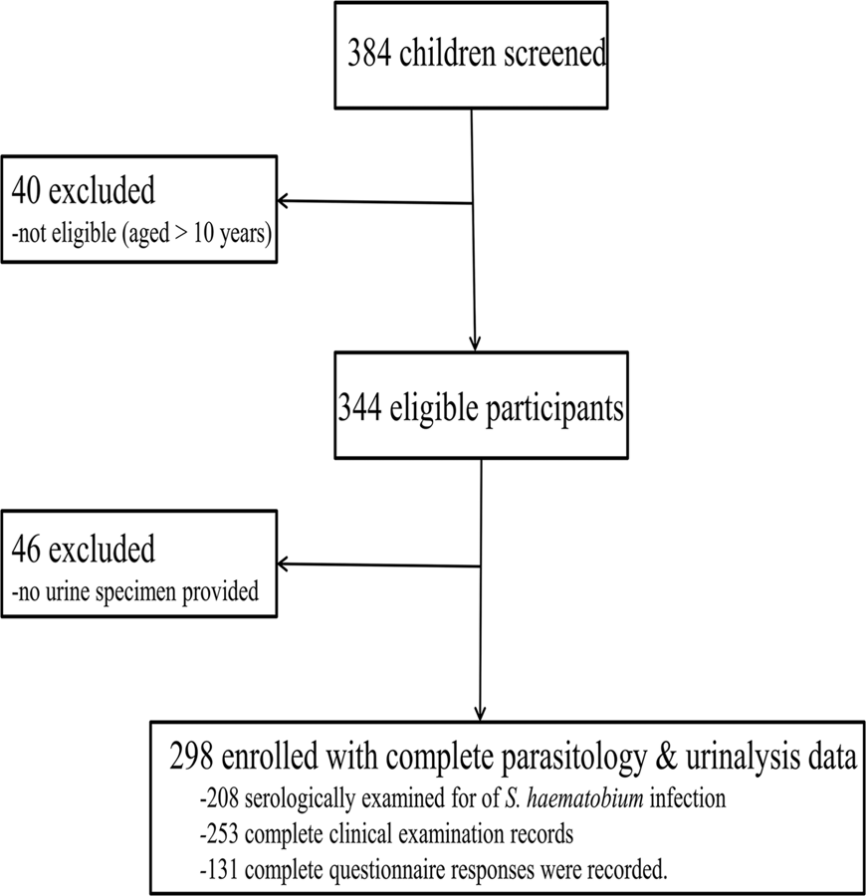
Flowchart indicating number of children enrolled in the study and excluded from the final analysis.

Our pre-study simulations revealed that a sample size of 129 children would provide 80.0% power to detect age group related differences in infection prevalence differences at α = 0.05, allowing for 5.0% non-compliance loss. These sample size calculations were based on the expected overall *S*. *haematobium* infection prevalence of 40.0% (for 1–5 years) and 60.0% (for 6–10 years), with information obtained from preliminary studies conducted in the same study area. Our final sample sizes for variables of interest were sufficiently large for statistical analyses (Fig. 1).

#### Statistical analyses

Correlations between continuous variables were measured using the Pearson’s correlation coefficient (r). The phi-coefficient (φ) was used for dichotomous variables. The chi-square (χ^2^) test was used to determine associations between different markers of morbidity by sex, age-group or *S. haematobium* infection status. Multiple logistic regressions were used to investigate factors influencing the prevalence of schistosome-related morbidity. Each of the morbidity indicators was included as a response variable, with sex (male *vs*. female), age-group (1–5 *vs*. 6–10 years) and *S. haematobium* infection status (determined by parasitology or serology) or infection intensity (log_10_[egg count+1]-transformed) included as risk factors in the models. Two-way interaction effects were included in model building, however, none were found to be significant and hence were subsequently dropped from the final models used for inferences.

Non-metric multidimensional scaling (NMDS) was used to explore the variability in dipstick attributes between children. For an outline of the NMDS modelling steps (see S1 File Supporting Information which explains the algorithm steps followed in this study and the test statistics used to evaluate the NMDS models). Correlation coefficients were used to identify dipstick attributes contributing most to overall variability in schistosome-related morbidity. The proportion of variability explained by each of the NMDS axes was measured using the coefficient of determination (R^2^R^2^). The multi-response permutation procedure (MRPP) was used to test the null hypothesis of no significant differences between subgroups. For each pairwise comparison, the resultant test-statistic (T) was reported along with the corresponding p-value [19].

The risk of morbidity for each age group was estimated using prevalence ratios, where a prevalence ratio greater than one indicated a positive association between schistosome infection and presence of related morbidity. The Breslow-Day test with Tarone’s adjustment for small sample sizes [20], was used to assess whether the probability of detecting morbidity using the different diagnostic tools in infected children differed between 1–5 years and 6–10 years old children. The population attributable fraction, and attributable fraction infected were used to estimate the proportion of morbidity in the whole study population and among infected children that could be attributed to *S*. *haematobium* infection respectively, adjusting for the effects of sex and age group. Furthermore, these estimates were used to compare the utility of the different diagnostic tools for detecting schistosome-related morbidity. Approximate 95% confidence intervals were calculated using the method described elsewhere [21]. For meaningful interpretations, attributable fractions were only estimated for the morbidity markers with a prevalence ratio (PR) significantly greater than one.

Sample size calculations were performed using StatXact v.8 (Cystel Software Corp, Cambridge, MA, USA). The NMDS analysis was performed using PCORD 6.08 (MjM Software, Gleneden Beach, Oregon, USA). Statistical modelling and tests for associations were performed using SAS 9.3 (SAS Institute Inc., Cary, NC, USA). In all analyses, the level of significance was set at p<0.05.

## Results

### Demographics

298 children (1–5 years: n = 104, median = 4 years; 6–10 years: n = 194, median = 8 years) fulfilled the study criteria (Fig. 1), and these comprised of 142 (47.7%) males, and 156 (52.3%) females.

### Schistosome infection levels

The overall prevalence of *S*. *haematobium* infection determined by parasitological examination was 35.9% (95% CI: 30.4–41.4%). When looking at infection intensities, 28.9% (95% CI: 23.7–34.0%) and 7.0% (95% CI: 4.1–10.0%) of these children carried respectively light and heavy infection intensities according to the WHO categorizations [8]. Infection prevalence amongst primary children aged 6–10 years was 47.9% (95% CI: 40.8–55.0%), and was significantly higher (χ^2^ = 35.0; p<0.001) compared to infection prevalence of 13.5% (95% CI: 6.8–20.1%) observed in 1–5 years old children. However, there was no significant difference (χ^2^ = 0.5; p = 0.466) in the prevalence of infection between male and female children. Infection intensity increased significantly with age (r = 0.4; p<0.001), with the highest levels observed between the ages of 8–10 years. The prevalence of *S*. *haematobium* infection determined by serology was higher than that determined by egg counts in both age groups, 1–5 years: 52.9% (95% CI: 38.8–67.1%), and 6–10 years: 84.1% (95% CI: 78.3–89.9%).

### Urinary dipstick morbidity markers

**Fig 2:**
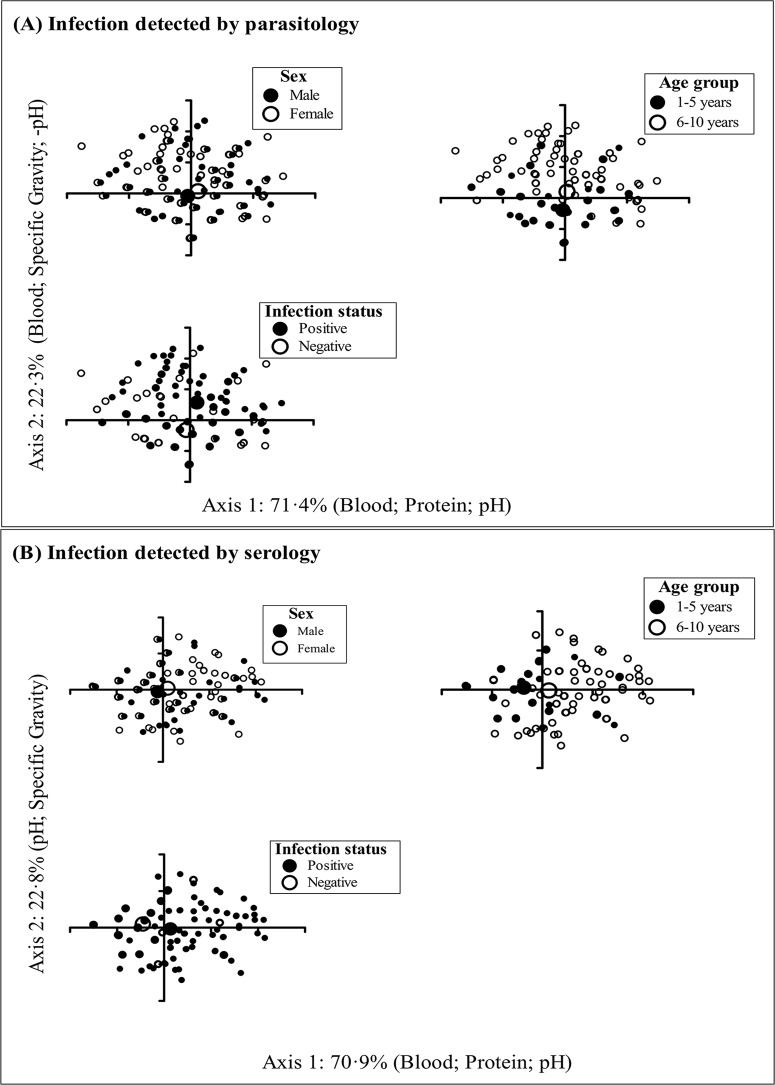
Non-metric multidimensional scaling (NMDS) ordination in 2-dimensional configurations by sex, age-group and *S. haematobium* infection status determined using parasitological (A) and serological diagnostic techniques (B). Subgroup centres are represented by the bigger closed (●), or open (○) points, and the distance between these centres is proportional to the level of dissimilarities between subgroups.

Dipstick-detected microhaematuria and proteinuria, contributed most to the observed variability in morbidity among children (taking into account urine’s physical characteristics, pH and specific gravity), as indicated by the strong correlations (see S1 Table). The variability of morbidity differed significantly between *S*. *haematobium* egg negative and positive children (*T* = -50.7; p<0.001) and between the two age groups (*T* = -19.3; p<0.001), however there were no differences by sex (*T* = -1.5; p = 0.089). Furthermore, the observed differences were evident from the large NMDS ordination output distances between the respective subgroup centres shown in Fig. 2. Based on the serological diagnosis of infection, significant differences were also observed by infection status (*T* = -14.0; p<0.001), age group (*T* = -6.5; p<0.001), but not by sex (*T* = -2.5; p = 0.068). In addition, microhaematuria and proteinuria alone explained about two-thirds of the overall variability due to differences between infected and uninfected children (detected by either parasitology or serology).

### Observed morbidity prevalence

**Fig 3:**
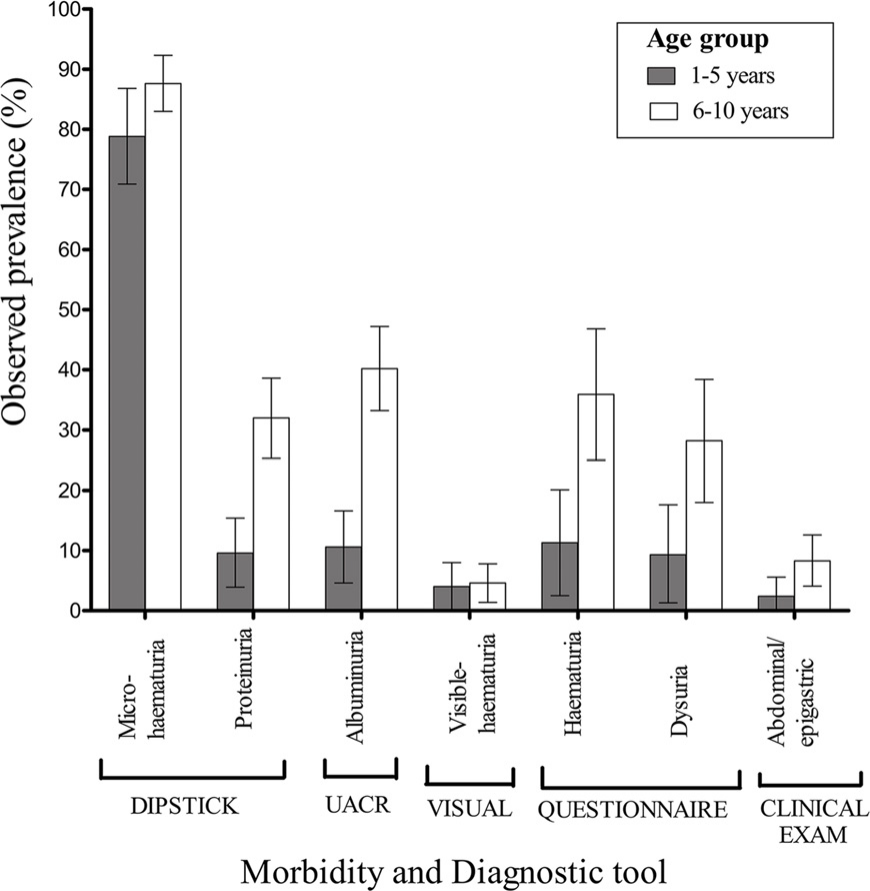
Observed prevalences of morbidity by age group, assessed using different diagnostic tools. Error bars indicate the 95% confidence intervals.

The prevalence of dipstick microhaematuria was higher than questionnaire-reported haematuria, which in turn was also higher than visible haematuria (Fig. 3). The morbidity prevalence results illustrated in Fig. 3 revealed that children aged 6–10 years tended to report morbidity more frequently compared to parents/guardians of 1–5 years old children. In addition, albuminuria (detected by UACR) and dipstick proteinuria were observed in both age groups as shown in Fig. 3. A positive association of albuminuria with microhaematuria (*φ* = 0.2, p = 0.002), or proteinuria (*φ* = 0.4; p<0.001) was observed. In comparison to other diagnostic techniques investigated in this study, visual urine inspection, and clinical examination detected the least number of morbidity cases (Fig. 3).

### Schistosome infection versus morbidity prevalence

Results of multiple logistic regression analyses revealed a significant positive association (in order of strength of the association) of visible haematuria, albuminuria, microhaematuria, and proteinuria with *S*. *haematobium* infection detected by parasitology as shown in Table 1. In addition, *S*. *haematobium* infection prevalence determined by serology was also found to be significantly associated with albuminuria and proteinuria, adjusting for the effects of sex and age group (Table 1). Significant increases in prevalence of albuminuria (Odds ratio (OR) = 5.5; p<0.001), visible haematuria (OR = 4.7; p<0.001), microhaematuria (OR = 3.4; p = 0.005), and proteinuria (OR = 3.3; p<0.001) with infection intensity were observed. However, no significant associations between infection intensity and questionnaire-reported haematuria and dysuria, or clinical examination detected morbidity were found.

**Table 1:** Multiple logistic regression odds ratios (OR) to investigate factors associated with the prevalence of morbidity assessed using different diagnostic tools.

			Infection detected by parasitology		Infection detected by serology	
Type of morbidity	Diagnostic tool used	Variable	OR (95% CI)	p	OR (95% CI)	p
Microhaematuria	Dipstick	Sex (M *vs*. F)	1.8 (0.9–3.4)	0.089	**2.7 (1.1–6.7)**	**0**.**031**
		Age group (1–5 *vs*. 6–10 years)	1.2 (0.6–2.4)	0.563	1.4 (0.5–3.9)	0.472
		Infection status (negative *vs*. positive)	**3.4 (1.5–7.9)**	**0.005**	0.9 (0.3–2.7)	0.902
Proteinuria	Dipstick	Sex (M *vs*. F)	1.2 (0.6–2.1)	0.594	1.2 (0.6–2.3)	0.564
		Age group (1–5 *vs*. 6–10 years)	**2.5 (1.2–5.5)**	**0.019**	2.0 (0.8–4.9)	0.145
		Infection status (negative *vs*. positive)	**3.3 (2.2–5.0)**	**<0.0001**	**4.5 (1.5–13.6)**	**0.007**
Albuminuria	UACR	Sex (M *vs*. F)	0.8 (0.4–1.5)	0.528	0.8 (0.4–1.5)	0.481
		Age group (1–5 *vs*. 6–10 years)	**3.1 (1.5–6.7)**	**0.004**	**3.4 (1.3–8.5)**	**0.011**
		Infection status (negative *vs*. positive)	**5.5 (3.4–8.9)**	**<0.0001**	**33.9 (4.5–254.0)**	**0.001**
Haematuria^a^	Visual inspection	Sex (M *vs*. F)	1.1 (0.3–3.6)	0.876	1.4 (0.3–5.9)	0.690
		Age group (1–5 *vs*. 6–10 years)	0.5 (0.1–1.9)	0.299	1.0 (0.2–5.3)	0.991
		Infection status (negative *vs*. positive)	**7.8 (1.8–34.4)**	**0.007**	-	-
Haematuria	Questionnaire	Sex (M *vs*. F)	1.0 (0.5–2.4)	0.931	1.6 (0.6–4.2)	0.349
		Age group (1–5 *vs*. 6–10 years)	**3.9 (1.4–10.8)**	**0.009**	**5.5 (1.1–27.6)**	**0.037**
		Infection status (negative *vs*. positive)	1.4 (0.6–3.3)	0.443	2.1 (0.4–11.2)	0.385
Dysuria	Questionnaire	Sex (M *vs*. F)	0.6 (0.3–1.6)	0.325	0.6 (0.2–1.4)	0.223
		Age group (1–5 *vs*. 6–10 years)	**4.1 (1.3–12.6)**	**0.013**	2.5 (0.7–9.1)	0.168
		Infection status (negative *vs*. positive)	1.0 (0.4–2.4)	0.926	1.6 (0.4–6.9)	0.531
Abdominal/	Clinical exam	Sex (M *vs*. F)	0.9 (0.3–2.5)	0.826	1.2 (0.4–3.5)	0.788
epigastric^b^		Age group (1–5 *vs*. 6–10 years)	-	-	-	-
		Infection status (negative *vs*. positive)	0.9 (0.3–2.6)	0.882	1.2 (0.3–4.4)	0.821

Significant effects (p<0.05) are shown in bold.

^a^OR not adjusted for serological infection status;

^b^OR not adjusted for age group effect.

### Morbidity attributable to *S. haematobium* infection

**Fig 4:**
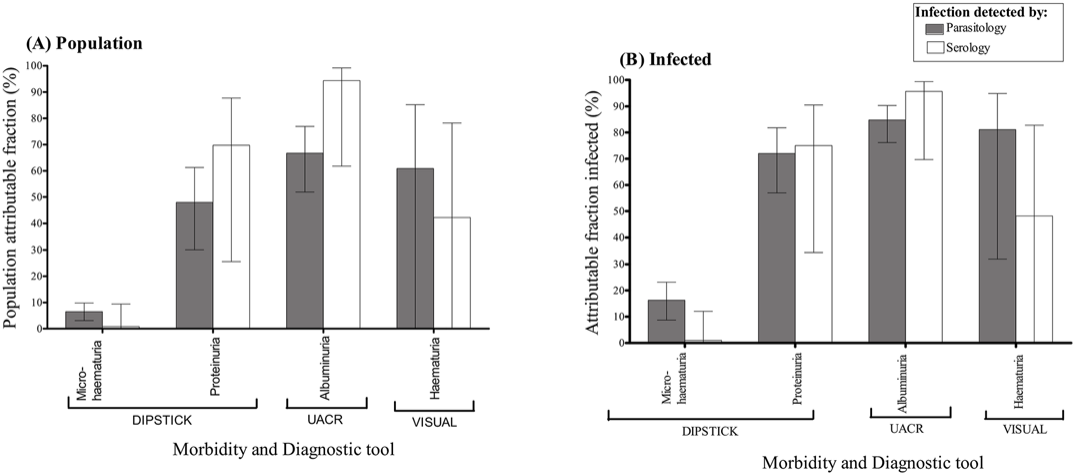
Estimated proportion of morbidity attributable to *S*. *haematobium* infection. (A) Population attributable fraction, (B) Attributable fraction infected.

Since the morbidity markers are not specific to schistosomes but are general markers of different physiological and biochemical processes, we went further to determine how much of the morbidity was attributable to schistosome infection. There was no significant difference in the estimated probability of detecting morbidity between 1–5 years and 6–10 years old children using each of the diagnostic tools (Table 2). In addition, from Table 2, it was observed that morbidity detected by dipsticks (microhaematuria and proteinuria), UACR (albuminuria), and urine inspection (visible haematuria) had prevalence ratios significantly greater than one. Clinical examination detected morbidity had the lowest prevalence ratio (Table 2). Furthermore, the results indicated that albuminuria was the dominant marker of schistosome attributable morbidity at population level, as well as amongst infected children (Fig. 4). Proteinuria and visible haematuria were also found to be highly attributable to schistosome infection among infected children. Although a high crude prevalence of microhaematuria was observed initially, the analyses revealed that a relatively small proportion of microhaematuria was attributed to *S*. *haematobium* infection (Fig. 4). The attributable fractions among infected children estimated by age group strata (see S1 Fig) showed a similar trend to the overall estimated attributable fractions noted above.

**Table 2:** Estimates of prevalence ratios (PR) weighted by age group for each of the morbidity markers assessed using different diagnostic tools and test for homogeneity of the probability of detecting morbidity in infected children.

		Infection detected by parasitology			Infection detected by serology		
Type of morbidity	Diagnostic tool used	PR (95% CI)	χ^2^-statistic	p	PR (95% CI)	χ^2^-statistic	p
Microhaematuria	Dipstick	**3.3 (1.4–7.9)**	1.4	0.231	0.9 (0.5–2.6)	0.4	0.509
Proteinuria	Dipstick	**1.5 (1.2–1.8)**	0.2	0.666	**1.3 (1.1–1.5)**	2.5	0.114
Albuminuria	UACR	**2.4 (1.9–3.1)**	0.01	0.927	**1.7 (1.4–1.9)**	0.3	0.571
Haematuria	Visual inspection	**1.1 (1.0–1.2)**	0.002	0.989	**1.5 (1.3–1.7)**	^a^	^a^
Haematuria	Questionnaire	1.1 (0.8–1.5)	5.1	0.024	1.1 (0.9–1.5)	1.5	0.225
Dysuria	Questionnaire	1.0 (0.8–1.3)	3.1	0.078	1.1 (0.8–1.6)	5.5	0.017
Abdominal/epigastric	Clinical exam	1.0 (0.3–2.9)	3.0	0.081	1.0 (0.9–1.1)	^a^	^a^

Comparisons between preschool-aged (1–5 years) versus primary school-aged (6–10 years) children. Prevalence ratios significantly higher than 1 are shown in bold.

^a^Test statistic could not be computed.

## Discussion

Until recently, most schistosome control programmes in Africa aimed at reducing development of severe morbidity and improving child health have focused on regular school-based deworming strategies, targeting children above five years old [22–24]. By focusing treatment upon the school-aged population, children of preschool-age have been previously neglected in terms of research and control [25]. Consequently, less is known about the levels of schistosome-related morbidity in this age-group. Furthermore, research studies evaluating the performance of the current POC markers of schistosome-related morbidity in children aged five years and below are still limited [11]. Estimation of disease burden due to schistosome infections in children has been further complicated by the fact that signs and symptoms commonly associated with schistosomiasis can also be due to other causes [26]. In the absence of a gold standard POC morbidity diagnostic technique, several methods have been used in studies from different endemic settings in older children (≥6 years) and adult populations [5]. Our study focused on the tools used in the field; the WHO approved questionnaire-based reporting of haematuria and dysuria, clinical examination by qualified clinicians, routinely used dipstick tests measuring several urine attributes, and UACR (for detecting albuminuria) which has previously been evaluated for schistosome morbidity detection [8]. We investigated how these tools performed in preschool-aged children (1–5 years) compared to primary school-aged children (6–10 years), who are the current targets of schistosome control programmes.

Our study revealed that children of the two age groups carried quantifiable levels of morbidity as determined by these different diagnostic tools. This finding is in accordance with a recent epidemiological study by Sacko and colleagues [27] who reported significant prevalence of urinary pathology in endemically exposed children. Of the several urine attributes tested using dipsticks, microhaematuria and proteinuria were significantly associated with *S*. *haematobium* infection, as it has been previously reported in several other studies [28–30]. A high proportion of children aged 5 years and below presented with microhaematuria in this study. More interestingly, the current study demonstrated that the performance of each of the different POC diagnostic tools for detecting morbidity did not differ between preschool and primary school-aged children infected with *S*. *haematobium*. These findings are important for planning of future interventions as they provide evidence that children ≤5 years can be effectively screened for praziquantel treatment using the available POC diagnostic tools applicable to older children and adult populations in the field [27,31].

Since the physical and biological features determined by these diagnostics can arise due to several conditions [32,33], we determined how much of the proportion of morbidity was attributed to *S*. *haematobium* infection. Based on the results of prevalence ratios and attributable fractions, UACR was identified as the most reliable tool for detecting schistosome-related morbidity, followed by dipsticks, visual urine inspection, questionnaires and lastly clinical examination. In addition, prevalence of albuminuria determined using UACR was positively associated with presence of microhaematuria and proteinuria detected by dipsticks. This finding suggests that these indicators used in combination can be a better predictor of the presence of urinary tract morbidity due to *S*. *haematobium* infection in children than using one test parameter alone, and thereby facilitating effective and timely interventions. The utility of albuminuria as a valuable indicator of schistosome-related morbidity in our study corroborates earlier findings in school-aged children by Sousa-Figueiredo and colleagues [34].

Although the proportion of children with visible haematuria was low in this study, it was noted that *S*. *haematobium* egg-positive children were eight times more likely to present with visible haematuria compared to egg-negative children. In addition, all children with visible haematuria were positive for *S*. *haematobium* infection detected using the serological diagnostic test. The majority of children in this study carried light infections, and this could explain the observed low prevalence of visible haematuria [35].

Since *S*. *haematobium* infection in endemic areas can easily be inferred from presence of blood in urine, questionnaire responses about recent/current presence of haematuria and dysuria can be used to assess schistosome-related morbidity. Our study showed some level of bias in the reporting of haematuria and dysuria between preschool-aged children, where the answers were provided by the parents/guardians and primary school-aged children, who responded to the questions themselves. One theoretical explanation for these observations could be that children easily mistook concentrated urine as blood in urine, but less likely so by adults and hence resulting in the overestimation of the prevalence of reported morbidity amongst the 6–10 years old children. These results therefore need to be interpreted with caution.

Physical clinical markers of morbidity were least attributable to schistosome infection, as previously mentioned. Our findings are consistent with a recent study by Agnew-Blais and colleagues [36], who also reported inadequacy of the physical examination method for assessing schistosome-related pathology in school-aged populations.

Nevertheless, there are some limitations when interpreting the results of our study. Firstly, given that approximately 30% of our study participants were not characterized for *S*. *haematobium* infection using the more sensitive serological diagnostic technique, caution must be applied when extrapolating the study findings. Secondly, in our stratified analysis the sample size was too limited to give precise estimates of schistosome-related morbidity prevalence measured using different markers; these results should be interpreted with caution.

### Conclusions

Our results confirm that schistosome infection in preschool children does result in significant morbidity. These findings are in agreement with recent studies on *S*. *mansoni* in Uganda [11] and *S*. *haematobium* in Malawi [37], reiterating the need for anthelminthic treatment in preschool children. This study has gone further to identify morbidity diagnostics with large fractions attributable to schistosome infection, highlighting detection of albuminuria as the best choice for rapid assessment of morbidity attributed to *S*. *haematobium* infection in children in the field. Finally the study showed that in *S*. *haematobium* endemic areas, preschool-aged children can be effectively screened for schistosome-related morbidity using the same diagnostic tools applicable to primary school-aged children and adult populations These findings are of clinical and public health importance, as these tools can be used to identify affected individuals or subgroups, thereby facilitating focused and timely delivery of treatment, as well as evaluate the effectiveness of interventions for improved control.

## Supporting Information

S1 Table Table that displays the Non-metric multidimensional scaling (NMDS) correlations (r) between urinary dipstick attributes and the two ordination axes. (DOC)

S1 Fig Figure that illustrates the proportion of morbidity attributable to *S. haematobium* infection detected by parasitology, estimated by age group strata. (TIF)

S1 File Text that explains the NMDS modelling steps used in the study. (DOC)

S2 File STROBE Checklist. (DOC)
