# Supplementary material for: Identifying and Evaluating Field Indicators of Urogenital Schistosomiasis-Related Morbidity in Preschool-Aged Children
Source: PLoS Negl Trop Dis. 2015 Mar 20;9(3):e0003649. doi: 10.1371/journal.pntd.0003649 (PMC4368198; doi:10.1371/journal.pntd.0003649)
Supplement: S1 Table — (DOC) [file pntd.0003649.s001.doc]

**Table 1.** **Non-metric multidimensional scaling (NMDS) correlations (r) between urinary dipstick attributes and the two ordination axes.** The coefficient of determination (R^2^) indicates the percentage of the overall variability explained. Strong correlations (absolute *r* ≥ 0.50) are presented in bold.

|  | **NMDS Axis 1 (71.4%)** | | **NMDS Axis 2 (22.3%)** | |
| --- | --- | --- | --- | --- |
| **Dipstick marker** | **r** | **R^2^ (%)** | **r** | **R^2^ (%)** |
| Blood | **-0**.**77** | 59.3 | **0**.**51** | 26.0 |
| pH | **-0**.**65** | 42.3 | **-0**.**68** | 46.2 |
| Specific gravity | 0.37 | 13.7 | **0**.**50** | 25.0 |
| Protein | **-0**.**79** | 62.4 | 0.01 | 0.01 |
| Leucocytes | -0.20 | 4.0 | 0.08 | 0.6 |
| Nitrite | -0.13 | 1.7 | 0.28 | 7.8 |
