# Supplementary figures and images for: Identifying and Evaluating Field Indicators of Urogenital Schistosomiasis-Related Morbidity in Preschool-Aged Children
Source: PLoS Negl Trop Dis. 2015 Mar 20;9(3):e0003649. doi: 10.1371/journal.pntd.0003649 (PMC4368198; doi:10.1371/journal.pntd.0003649)

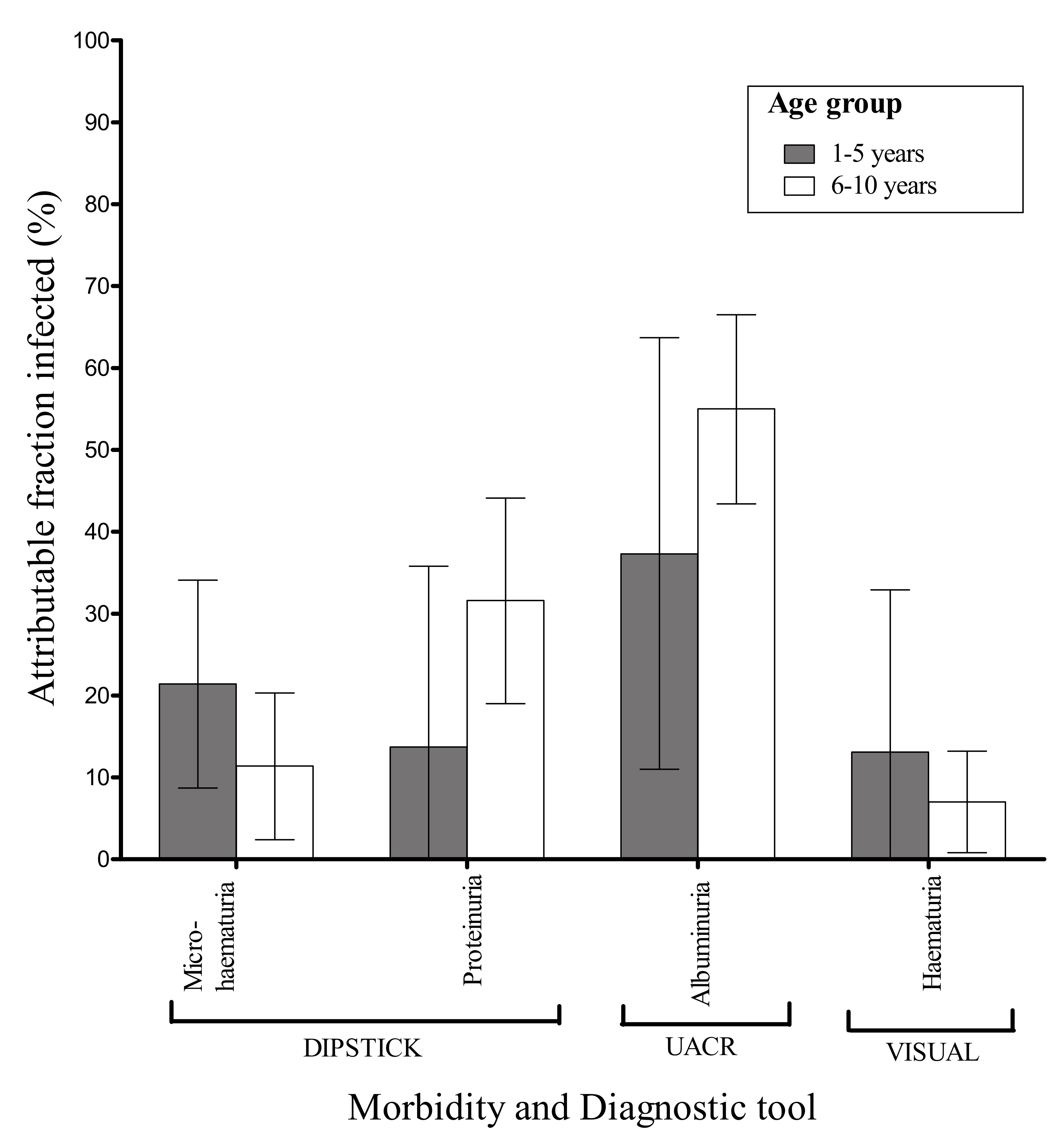

Supplement: S1 Fig — (TIF) [file pntd.0003649.s002.tif]
