# Supplementary material for: Identifying and Evaluating Field Indicators of Urogenital Schistosomiasis-Related Morbidity in Preschool-Aged Children
Source: PLoS Negl Trop Dis. 2015 Mar 20;9(3):e0003649. doi: 10.1371/journal.pntd.0003649 (PMC4368198; doi:10.1371/journal.pntd.0003649)
Supplement: S1 File — (DOC) [file pntd.0003649.s003.doc]

# Supplementary material

## Non-metric multidimensional scaling (NMDS) modelling steps

Non-metric multidimensional scaling (NMDS), a multivariate non-parametric data reduction technique [1], was used to identify urinary dipstick markers that contributed most to the differences in schistosome-related morbidity observed in this population. The NMDS model runs were based on Bray-Curtis distances with no penalty on handling ties, performed using PCORD 6.08 (MjM Software, Gleneden Beach, Oregon, USA), following the algorithm steps described in Bourke and colleagues [2]. Five hundred iterations with real data were made, with 15 iterations used to evaluate model stability based on a stability criterion of 0.000001. An initial 6-dimensional NMDS was performed in search of an optimal number of axes that best represented the variation in the data set. Using a scree plot, it was determined that a two-dimensional NMDS ordination with final instability of 0.0 and stress value (goodness of fit) of 8.5 was adequate to account for most of the observed variability. A multi-response permutation procedure (MRPP) test was performed in R 3.0.1 (R Development Core Team, Vienna, Austria) to assess differences in the NMDS output by sex, age group and *S. haematobium* infection status. A total of 1, 000 permutations of the data were executed to test for significance. Pairwise comparisons between subgroups were conducted using a combination of MRPP statistics, namely: the T-statistic, an equivalent of the Student’s t-test describing the between-group separation, measure of effect size, A, indicating the chance-corrected within-group similarity and the p-value representing the probability of having obtained as low an average within group similarity as actually observed [3]. Significant group differences implied that children belonging to one subgroup were more similar in terms of the measured urine attributes than they would be expected if they had belonged to the other group. In addition, similarity percentage (SIMPER) analysis was conducted to further assess the individual contribution of each of the urinary dipstick attributes to the overall dissimilarities between subgroups. Plots of the resultant two ordination axes by subgroups were used to reflect patterns of variability in the original multivariate dipstick attributes among children as captured in the NMDS. The distance between points in the ordination space is proportional to the underlying distance measure between these points [3].

###### References

1. Cox TF, Cox MAA. Multidimensional Scaling; Cox DR, Isham V, Keiding N, Louis T, Reid N et al., editors. London: Chapman & Hall/CRC; 2001.

2. Bourke CD, Mutapi F, Nausch N, Photiou DM, Poulsen LK, et al. *Trichuris suis* ova therapy for allergic rhinitis does not affect allergen-specific cytokine responses despite a parasite-specific cytokine response. Clin Exp Allergy. 2012;42: 1582-1595.

3. Peck JE. Multivariate Analysis for Community Ecologists: Step-by-Step using PC-ORD. Gleneden Beach, Oregon: MjM Software Design; 2010.
