# Supplementary material for: Identifying and Evaluating Field Indicators of Urogenital Schistosomiasis-Related Morbidity in Preschool-Aged Children
Source: PLoS Negl Trop Dis. 2015 Mar 20;9(3):e0003649. doi: 10.1371/journal.pntd.0003649 (PMC4368198; doi:10.1371/journal.pntd.0003649)
Supplement: S2 File — (DOC) [file pntd.0003649.s004.doc]

STROBE Statement—Checklist of items that should be included in reports of ***cross-sectional studies***

|  | Item No | Recommendation |
| --- | --- | --- |
| **Title and abstract** | 1 | (*a*) Indicate the study’s design with a commonly used term in the title or the abstract |
|  |  | **Indicated (Methods/Findings)** |
|  |  | (*b*) Provide in the abstract an informative and balanced summary of what was done and what was found  **Provided (Method/Findings and Conclusions/Significance)** |
| Introduction | | |
| Background/rationale | 2 | Explain the scientific background and rationale for the investigation being reported  **Explained in paragraphs 1-3.** |
| Objectives | 3 | State specific objectives, including any prespecified hypotheses  **Provided (Paragraph 3)** |
| Methods | | |
| Study design | 4 | Present key elements of study design early in the paper  **Provided (Sections: Study area, Participants)** |
| Setting | 5 | Describe the setting, locations, and relevant dates, including periods of recruitment, exposure, follow-up, and data collection  **Provided (Sections: Study area, Participants)** |
| Participants | 6 | (*a*) Give the eligibility criteria, and the sources and methods of selection of participants  **Provided (Sections: Study area, Participants)** |
| Variables | 7 | Clearly define all outcomes, exposures, predictors, potential confounders, and effect modifiers. Give diagnostic criteria, if applicable  **Provided (Sections: Parasitology and Serology, Statistical Methods)** |
| Data sources/ measurement | 8* | For each variable of interest, give sources of data and details of methods of assessment (measurement). Describe comparability of assessment methods if there is more than one group  **Provided (Sections: Parasitology and Serology, Morbidity Measurement)** |
| Bias | 9 | Describe any efforts to address potential sources of bias  **Provided (Section: Participants). To be included in this study, participants had to meet the following criteria: (1) been lifelong residents of the study area, (2) had no prior history of antihelminthic treatment (assessed by questionnaires administered to parents/guardians for all children)** |
| Study size | 10 | Explain how the study size was arrived at  **Explained (Section: Statistical Methods, Subsection: Sample size Calculation)** |
| Quantitative variables | 11 | Explain how quantitative variables were handled in the analyses. If applicable, describe which groupings were chosen and why  **Provided (Sections: Parasitology and serology). Children were categorized as infected based on serology if their anti-egg IgM antibody levels were more than two standard deviations above the mean estimated from sera of negative controls** |
| Statistical methods | 12 | (*a*) Describe all statistical methods, including those used to control for confounding |
|  |  | (*b*) Describe any methods used to examine subgroups and interactions |
|  |  | (*c*) Explain how missing data were addressed |
|  |  | (*d*) If applicable, describe analytical methods taking account of sampling strategy |
|  |  | (*e*) Describe any sensitivity analyses  **All provided where applicable (Section: Statistical methods)** |
| Results | | |
| Participants | 13* | (a) Report numbers of individuals at each stage of study—eg numbers potentially eligible, examined for eligibility, confirmed eligible, included in the study, completing follow-up, and analysed  **Considered.** |
|  |  | (b) Give reasons for non-participation at each stage  **Considered (Section: Participants). The children were recruited into the study on voluntary basis and were free to withdraw at any time with no further obligation.** |
|  |  | (c) Consider use of a flow diagram  **Considered and provided (Fig. 1)** |
| Descriptive data | 14* | (a) Give characteristics of study participants (eg demographic, clinical, social) and information on exposures and potential confounders  **Considered (Results section: Demographics)** |
|  |  | (b) Indicate number of participants with missing data for each variable of interest  **Considered (Results section: Demographics and Fig. 1)** |
| Outcome data | 15* | Report numbers of outcome events or summary measures  **Considered** |
| Main results | 16 | (*a*) Give unadjusted estimates and, if applicable, confounder-adjusted estimates and their precision (eg, 95% confidence interval). Make clear which confounders were adjusted for and why they were included |
|  |  | (*b*) Report category boundaries when continuous variables were categorized |
|  |  | (*c*) If relevant, consider translating estimates of relative risk into absolute risk for a meaningful time period  **Considered, adjusting for confounders stated in Results subsections** |
| Other analyses | 17 | Report other analyses done—eg analyses of subgroups and interactions, and sensitivity analyses  **Considered. (Results section) Attribuatbel fraction infected estimated by age group, S3 Supplementary Figure** |
| Discussion | | |
| Key results | 18 | Summarise key results with reference to study objectives  **Considered** |
| Limitations | 19 | Discuss limitations of the study, taking into account sources of potential bias or imprecision. Discuss both direction and magnitude of any potential bias  **NA** |
| Interpretation | 20 | Give a cautious overall interpretation of results considering objectives, limitations, multiplicity of analyses, results from similar studies, and other relevant evidence  **Considered.** |
| Generalisability | 21 | Discuss the generalisability (external validity) of the study results  **Considered** |
| Other information | | |
| Funding | 22 | Give the source of funding and the role of the funders for the present study and, if applicable, for the original study on which the present article is based  **Considered.** |

*Give information separately for exposed and unexposed groups.

**Note:** An Explanation and Elaboration article discusses each checklist item and gives methodological background and published examples of transparent reporting. The STROBE checklist is best used in conjunction with this article (freely available on the Web sites of PLoS Medicine at http://www.plosmedicine.org/, Annals of Internal Medicine at http://www.annals.org/, and Epidemiology at http://www.epidem.com/). Information on the STROBE Initiative is available at www.strobe-statement.org.
